# Clinical Validation of the Comprehensive Complication Index in Colon Cancer Surgery

**DOI:** 10.3390/cancers13071745

**Published:** 2021-04-06

**Authors:** Nicolò Tamini, Davide Bernasconi, Lorenzo Ripamonti, Giulia Lo Bianco, Marco Braga, Luca Nespoli

**Affiliations:** 1 School of Medicine and Surgery, University Milano-Bicocca, 20900 Monza, Italy; davide.bernasconi@unimib.it (D.B.); l.ripamonti10@campus.unimib.it (L.R.); giulialobi@yahoo.it (G.L.B.); marco.braga@unimib.it (M.B.); luca.nespoli@unimib.it (L.N.); 2 ASST Ospedale San Gerardo, 20090 Monza, Italy

**Keywords:** Clavien–Dindo, Comprehensive Complication Index, CCI, colon cancer, postoperative complications

## Abstract

**Simple Summary:**

The results of this study showed a greater ability of the Comprehensive Complication Index if compared to the conventional Clavien–Dindo classification to predict hospital stay in colon cancer patients, particularly in patients with multiple postoperative complications. These results encourage the routine use of the Comprehensive Complication Index to grade postoperative complications in colonic surgery.

**Abstract:**

(1) Introduction: To date, the sensitivity of the Comprehensive Complication Index (CCI) in a homogeneous cohort of colonic resections for oncologic purposes has not been reported. The present study aims to compare the CCI with the conventional Clavien–Dindo classification (CDC) in colon cancer patients. (2) Methods: The clinical data of patients submitted to an elective colectomy for adenocarcinoma were retrieved from a prospectively maintained database. Postoperative complications and length of stay were reviewed, and CDC and CCI scores were calculated for each patient. The association of the CCI and the CDC with the length of stay, prolongation of stay and readmission rate were assessed and compared. (3) Results: The overall postoperative morbidity was 26.9%. In particular, 157 (20.4%) patients had more than one complication. A strong correlation between the two scoring systems was observed (r = 99.4%; 95%CI: 99.3–99.5%). In multivariate analysis, CCI had a higher predictive ability for all endpoints. Regarding subgroup analysis, the difference between the CCI and CDC was progressively increased when evaluating outcome measures in complicated and multi-complicated patients. (4) Conclusion: Both scoring systems adequately report the overall burden of postoperative complications. The CCI showed a greater ability than the CDC to predict hospital stay, particularly in patients with multiple postoperative complications.

## 1. Introduction

Although the perioperative mortality rate following elective colon cancer surgery has been significantly reduced over the past few years, postoperative morbidity remains a significant issue [1,2,3].

Moreover, there is growing evidence that postoperative complications not only have a direct impact on short-term outcomes but also impact systemic inflammatory response, which may adversely affect the oncological outcome [4,5,6].

Currently, postoperative complications constitute a universally accepted marker of surgical outcomes and quality of care; however, their expression often lacks clear standardization [7,8].

In 2004, Clavien, Dindo and their colleagues proposed a five-grade classification [9] that remains the most widely used scoring system for grading postoperative morbidity.

Since the score calculation is based on the most severe complication only, the Clavien Dindo classification (CDC) might underestimate the real burden of postoperative morbidity, especially in patients suffering multiple complications.

To overcome this potential limitation, the Comprehensive Complication Index (CCI), which integrates the severity of each postoperative complication, was recently proposed [10]. The CCI is calculated as the sum of all complications that are weighted for their severity. The final formula yields a continuous scale to rank the severity of any combination of complications from 0 to 100 in a single patient.

While this index has been validated in different or heterogeneous surgical procedures, clinical validation of the CCI in a homogeneous cohort of colon cancer patients has not been reported to date [11,12,13,14,15].

Therefore, the present study aims to compare the ability of the CCI and CDC in predicting length of stay, prolongation of stay and readmission in a large cohort of patients who underwent elective surgery for colon cancer.

## 2. Methods

The present study is reported according to the STROBE guidelines for the conducting and reporting of observational cohort studies [16].

### 2.1. Study Design

This is a retrospective analysis of a prospectively collected database including patients aged more than 18 years who underwent an elective colectomy for cancer at a tertiary referral university hospital between January 2010 and December 2018. 

Patients who underwent surgical procedure performed in an emergency setting were excluded.

All patients were managed according to the Enhanced Recovery after Surgery (ERAS^®^) pathway for colorectal surgery [17], particularly regarding certain core items such as routine antibiotic prophylaxis, restrictive fluids balance, postoperative nausea and vomiting prophylaxis, active warming, thromboembolic prophylaxis and early postoperative feeding.

### 2.2. Data Collection and Outcome Measures

Clinical data were retrieved from an institutional electronic spreadsheet. All oncologic patients for whom an elective colonic resection was performed were included. 

Preoperative data included demographics and patient comorbidities classified according to the Charlson Comorbidity Index [18] and American Society of Anesthesiologists (ASA) score [19].

Intraoperative data included the surgical procedure, surgical technique and operative time.

A side-to-side handsewn extracorporeal anastomosis was performed in the case of a right colectomy, while a stapled transanal end-to-end colorectal anastomosis was performed in the case of a left-side colectomy.

Postoperative complications were defined according to *a priori* defined criteria [20] and graded according to the CDC. Major complications were defined as CDC grade 3 or above.

All complications that occurred during hospitalization or within 30 days after discharge were considered relevant to the surgical procedure regardless of patient readmission.

Patients suffering from at least one complication were defined as complicated, while patients with more than one complication were defined as multi-complicated.

The CCI was calculated using an online calculator [21], by entering the complication data for each patient.

In case of doubt, each case was reassessed in a collegial clinical session to assign the most appropriate CDC and CCI score.

Postoperative length of stay (LOS) was evaluated from the time of the surgical procedure to the time of discharge. Prolonged LOS was defined as an inpatient hospital stay longer than the 75th percentile of postoperative LOS. Hospital readmission for any postoperative complication occurring within 30 days of discharge was recorded. 

The CDC and the CCI, as main outcome measures, were benchmarked and compared by evaluating their association with LOS, prolonged LOS and readmission rate.

### 2.3. Statistical Analysis

Patients’ characteristics were summarized using numbers and percentages for categorical variables and median with interquartile range for continuous variables. The distribution of CDC and CCI was described using boxplots, and the association between the two scores was evaluated using the Spearman correlation index.

Using quantile regression, we estimated two univariate models for the median LOS using either CDC or CCI as independent variables. We compared the performance of the two models using a proper goodness of fit statistic [22]. Two multivariate models adjusting for age, gender, Charlson Comorbidity index, ASA 3–4 vs. 1–2 and laparoscopy vs. open surgery were also fitted and compared. Furthermore, we evaluated the association between the complication scores and two binary endpoints: prolonged LOS and readmission. We fit univariate and multivariate (adjusting for the same aforementioned factors) logistic regression models. The Hosmer–Lemeshow goodness of fit test and the area under the roc curve (AUC) index were used to assess and compare the predictive performance of the models. The same procedures were employed to benchmark the CDC against the CCI in the subgroups of patients with at least one complication (complicated) and with more than one complication (multi-complicated).

All the analyses were performed using R (version 3.6.0).

## 3. Results

A total of 958 consecutive patients hospitalized for colonic cancer were extracted from our prospectively constructed database. Of these, 121 patients were excluded due to emergency procedures, and 52 patients dropped out due to palliative surgery without the removal of primary cancer.

A total of 770 patients were included in the final analysis. The characteristics of the study population are depicted in Table 1.

Right colectomy was performed in 506 (65.7%) patients, while 264 (34.3%) patients underwent left colectomy.

A successful laparoscopic procedure was performed in 378 (49.1%) patients, with an increasing rate over the years reaching 82% when considering the last five years of the study period.

Postoperative outcomes are reported in Table 2. 

Overall postoperative morbidity was 26.9%, while 157 (20.4%) patients had more than one complication. 

A major complication according to the CDC was observed in 7.4% of patients. The median postoperative length of stay was 8 days (IQR 7-11) and the 30-day readmission rate was 4.8%. 

**Figure 1:**
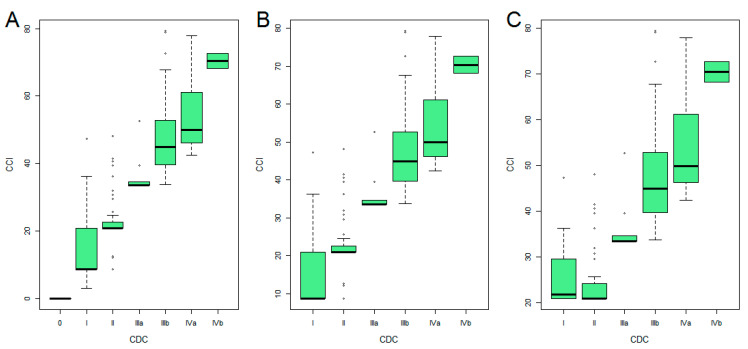
Distribution of CCI and CDC in the overall series (**A**), the subgroup of complicated patients (**B**) and the subgroup of patients with more than one complication (**C**).

Figure 1 reports the distribution of CCI and CDC in the overall series, the subgroup of complicated patients and the subgroup of patients with more than one complication, respectively.

The correlation between the two scoring systems was 78.8% (95%CI: 73.0–83.5%) in the subgroup of patients with at least one complication and 74.1% (95%CI: 66.1–80.4%) in the subgroup of patients with more than one complication.

A similar median CCI score was observed in patients graded as 3b and 4a according to the CDC (44.9 [IQR 39.7–52.7] and 49.9 [IQR 46.2–61.1], respectively).

Regarding the univariate analysis of the study population, both CDC and CCI showed a strong association with LOS, prolonged LOS and readmission; however, the CCI had a higher predictive ability for all endpoints (Table 3). The multivariate analysis, adjusted for age, gender, Charlson comorbidity index, ASA and procedure type, confirmed the aforementioned results (Table 3). 

Regarding univariate subgroup analysis, the difference in goodness of fit between the CCI and CDC progressively increased when evaluating LOS as an outcome measure in complicated (0.25 vs. 0.18, respectively) and multi-complicated patients (0.31 vs. 0.17, respectively). 

For the binary outcomes, prolonged LOS and readmission, an analogue increase in the difference of AUC values between CCI and CDC was observed in the two patient subgroups. Considering prolonged LOS, CCI showed a greater AUC compared to CDC in complicated patients (0.75 and 0.70, respectively) and patients with more than one complication (0.74 and 0.65, respectively).

Considering readmission as the outcome, the CCI showed a greater AUC compared to the CDC in complicated (0.72 and 0.65, respectively) and multi-complicated patients (0.67 and 0.60, respectively).

Once more, the aforementioned results were confirmed by the multivariate analysis.

## 4. Discussion

The present study validated the CCI in a monocentric homogeneous population of patients submitted to colonic surgery for cancer. Notably, the CCI had a greater ability than the CDC to predict hospital stay, particularly in patients with multiple postoperative complications.

At present, the CDC is widely used for assessing and reporting postoperative morbidity. It focuses on the most severe form of complication, which is graded according to the invasiveness of the required treatment.

This system has been extensively validated and gained wide application across different fields of surgery due to its ease and reproducibility.

Nevertheless, the exclusive evaluation of the most severe postoperative complication constitutes an intrinsic limitation in reporting the overall morbidity burden of a surgical procedure.

The CCI system considers each complication, resulting in a score ranging from 0 to 100, which represents the overall morbidity burden of a single surgical procedure.

A better correlation of the CCI with a postoperative hospital stay when compared to the CDC was observed in a series of patients who underwent esophagectomy [23] and gastrointestinal surgery [24]. While few studies have focused on colorectal surgery in this context, no data on a large series of patients who underwent elective colonic surgery for cancer have been published to date.

Notably, Slankamenac et al. [25] externally validated the CCI on a multicenter randomized controlled trial including 62 patients submitted to left colonic resection for perforated diverticulitis. A significant association between the CCI and hospital and intensive care unit stay was found. Moreover, Van Rooijen et al. [26] found that a CCI ≥ 20 was associated with a longer hospital stay in a cohort of 139 patients who underwent mixed colorectal procedures.

Furthermore, the CCI was identified as an independent predictor of readmission following colonic or rectal surgery in a cohort of 284 patients [27].

To benchmark the CDC and CCI on a homogeneous population, patients undergoing rectal surgery or emergency colonic surgery were excluded from the present study. 

Focusing on a homogeneous study population with similar surgical complexity and overall postoperative morbidity burden is a fundamental prerequisite to clarify the role of CCI in predicting postoperative outcome indicators. 

As in previous studies [12,13], the CDC and CCI have been validated by exploring their association with postoperative stay, prolonged stay and readmission rate.

However, differently from other studies [23], reoperation and intensive care unit stay have not been considered as endpoints in our study, since both are determinants in the CDC grading process; therefore, their inclusion in regression models would result in bias.

Our study population has a high mean age and comorbidity rate, while laparoscopic surgery was performed in approximately half of the patients, and 40% of patients had stage III–IV colon cancer.

In our experience, all of these factors might have a relevant impact on morbidity rate and severity, length of stay and readmission [28,29,30]. As such, this should be considered when interpreting the results. When the analysis included all patients with complications, the CCI had a slightly higher ability to predict LOS and prolonged LOS when compared to the CDC, which is similar to previous results in different series of patients [12,13,23,31].

In the present series, 75% of eventful patients had more than one complication reflecting the complexity of both their preoperative characteristics and surgical procedures. 

Limiting the analysis to the subgroup of patients with more than one complication, the CCI showed a stronger association with hospital stay when compared to the CDC.

The similar ability of the two scoring systems in predicting hospital readmission could be due to the small proportion of observed events. 

Again, CCI better predicts readmission than CDC in the subgroup of patients with more than one complication.

The low proportion of patients with major complications could explain the small difference in the predictive ability of both indexes. 

The retrospective design is a limitation of the present study; however, the prospective data collection and a priori defined criteria to identify each postoperative complication might mitigate the possible bias in CDC and CCI score calculation. 

The single-center study design could potentially decrease the generalizability of our results; however, it might simultaneously result in lower discharge criteria variability. Moreover, differences in postoperative treatment of surgical complications were minimized by the monocentric design of the study.

Notably, the large series of patients is a strength of the present study, mainly because it allowed selective analyses in patients with postoperative complications and the subgroup of patients with more than one complication.

Especially when the rate of uneventful patients is high, limiting analysis on the overall series may translate into unreliable results.

## 5. Conclusions

The CCI adequately reports the overall burden of postoperative complications in patients who underwent colonic resection for cancer. The CCI demonstrated a greater ability than the CDC to predict hospital stay, particularly in patients with multiple postoperative complications.

## Figures and Tables

**Table 1:** Characteristics of the study population.

	Overall (N = 770)	Complicated (N = 207)	Multi-Complicated (N = 157)
**Age** (median, IQR)	73 (64–79)	76 (67–81)	76 (67–81)
**Sex** (n, %)			
Male	389 (50.5)	121 (58.5)	90 (57.3)
Female	381 (49.5)	86 (41.5)	67 (42.7)
**Charlson Comorbidity Index** (median, IQR)	6 (4–7)	6 (5–8)	6 (5–8)
**Preoperative Hemoglobin** (g/dL; median, IQR)	11.9 (10.3–13.6)	11.5 (10.1–13.3)	11.4 (10.1–13.1)
**ASA score** (n, %)			
1	45 (5.8)	6 (2.9)	5 (3.2)
2	420 (54.5)	83 (40.1)	66 (42.0)
3	282 (36.6)	107 (51.7)	81 (51.6)
4	23 (3.1)	11 (5.3)	5 (3.2)
**Surgical procedure** (n, %)			
Left colectomy	264 (34.3)	70 (33.8)	52 (33.1)
Right colectomy	506 (65.7)	137 (66.2)	105 (66.9)
**Operative time** (minutes; median, IQR)	172 (140–215)	175 (135–222.5)	165 (135–220)
**Laparoscopy** (n, %)	378 (49.1)	91 (44.0)	69 (43.9)
**Stage** (n, %)			
I–II	460 (59.7)	112 (54.1)	89 (56.7)
III	251 (32.6)	78 (37.7)	54 (34.4)
IV	59 (7.7)	17 (8.2)	14 (8.9)

**Table 2:** Outcomes of the study population.

	Overall (N = 770)	Complicated (N = 207)	Multi-Complicated (N = 157)
**Type of complication** (n, %)			
**Anastomotic leakage**	27 (3.5)	27 (13.0)	27 (17.2)
**Surgical site infection**	44 (5.7)	44 (21.3)	35 (22.3)
**Intraperitoneal abscess**	13 (1.7)	13 (6.3)	13 (8.3)
**Respiratory complication**	29 (3.8)	29 (14.0)	26 (16.6)
**Cardiovascular complication**	27 (3.5)	27 (13.0)	18 (11.5)
**Urinary tract infection**	14 (1.8)	14 (6.8)	8 (5.1)
**Neurological complication**	12 (1.6)	12 (5.8)	4 (2.5)
**Perioperative transfusion**	66 (8.6)	66 (31.9)	60 (38.2)
**Others**	46 (6.0)	46 (22.2)	37 (23.6)
**Postoperative LOS** (days; median, IQR)	8 (7–11)	12 (9–18)	13 (9–21)
**Prolonged LOS** (n, %)	167 (21.7)	131 (63.3)	111 (70.7)
**CDC** (n, %)			
0	563 (73.1)	-	-
1	53 (6.9)	53 (25.6)	20 (12.7)
2	97 (12.6)	97 (46.9)	80 (51.0)
3a	9 (1.2)	9 (4.3)	9 (5.7)
3b	35 (4.5)	35 (16.9)	35 (22.3)
4a	11 (1.4)	11 (5.3)	11 (7.0)
4b	2 (0.3)	2 (1.0)	2 (1.3)
**CCI** (median, IQR)	0 (0–8.7)	20.9 (20.9–36.2)	29.6 (20.9–40.6)
**30 days readmission** (n, %)	37 (4.8)	19 (9.2)	18 (11.5)

**Table 3:** Univariate and multivariate analysis for LOS, prolonged LOS and readmission.

	OVERALL						COMPLICATED						MULTI-COMPLICATED					
LOS	CCI			CDC			CCI			CDC			CCI			CDC		
Analysis	Median LOS Change per 10 Units Increment (95% CI)		Model Goodness of Fit	Median LOS Change per Category Increment (95% CI)		Model goodness of fit	Median LOS Change per 10 Units Increment (95% CI)		Model goodness of fit	Median LOS Change per Category Increment (95% CI)		Model Goodness of Fit	Median LOS Change per 10 Units Increment (95% CI)	Model Goodness of Fit		Median LOS Change per Category Increment (95% CI)	Model Goodness of Fit	
**Univariate**	2.392 (1.909–2.648)		0.186	2.750 (1.435–3.858)		0.160	4.068 (3.178–5.000)		0.245	4.667 (2.964–5.846)		0.179	5.960 (4.284–6.665)	0.312		5.0 (3.214–7.680)	0.168	
**Multivariate**	2.448 (2.038–2.723)		0.169	2.715 (2.306–3.207)		0.149	3.997 (3.059–5.124)		0.219	3.985 (3.335–5.287)		0.162	6.064 (4.550–6.594)	0.299		4.904 (3.526–6.315)	0.166	
**Prolonged LOS**	**CCI**			**CDC**			**CCI**			**CDC**			**CCI**			**CDC**		
**Analysis**	**OR per 10 Units Increment (95% CI)**	**Hosmer-Lemeshow Test *p*-Value**	**AUC**	**OR per Category Increment (95% CI)**	**Hosmer-Lemeshow Test *p*-Value**	**AUC**	**OR per 10 Units Increment (95% CI)**	**Hosmer-Lemeshow Test *p*-Value**	**AUC**	**OR per Category Increment (95% CI)**	**Hosmer-Lemeshow Test *p*-Value**	**AUC**	**OR per 10 Units Increment (95% CI)**	**Hosmer-Lemeshow Test *p*-Value**	**AUC**	**OR per Category Increment (95% CI)**	**Hosmer-Lemeshow Test *p*-Value**	**AUC**
**Univariate**	2.513 (2.150–2.937)	0.988	0.771	2.954 (2.457–3.551)	1	0.766	2.047 (1.547–2.709)	0.605	0.752	2.284 (1.627–3.206)	1	0.700	2.044 (1.388–3.011)	0.861	0.736	1.824 (1.277–2.605)	0.994	0.646
**Multivariate**	2.418 (2.040–2.867)	0.064	0.837	2.795 (2.289–3.412)	0.215	0.830	1.936 (1.431–2.619)	0.952	0.801	2.056 (1.432–2.953)	0.934	0.782	1.920 (1.277–2.889)	0.397	0.797	1.725 (1.172–2.539)	0.603	0.772
**Readmission**	**CCI**			**CDC**			**CCI**			**CDC**			**CCI**			**CDC**		
**Analysis**	**OR per 10 Units Increment (95% CI)**	**Hosmer-Lemeshow Test *p*-Value**	**AUC**	**OR per Category Increment (95% CI)**	**Hosmer-Lemeshow Test *p*-Value**	**AUC**	**OR per 10 Units Increment (95% CI)**	**Hosmer-Lemeshow Test *p*-Value**	**AUC**	**OR per Category Increment (95% CI)**	**Hosmer-Lemeshow Test *p*-Value**	**AUC**	**OR per 10 Units Increment (95% CI)**	**Hosmer-Lemeshow Test *p*-Value**	**AUC**	**OR per category increment (95% CI)**	**Hosmer-Lemeshow Test *p*-Value**	**AUC**
**Univariate**	1.456 (1.255–1.688)	1	0.657	1.507 (1.241–1.830)	1	0.648	1.522 (1.184–1.957)	0.181	0.720	1.444 (1.021–2.044)	0.925	0.651	1.444 (1.088–1.916)	0.382	0.673	1.291 (0.884–1.886)	0.958	0.604
**Multivariate**	1.482 (1.258–1.745)	0.606	0.691	1.524 (1.232–1.886)	0.498	0.693	1.808 (1.321–2.475)	0.727	0.828	1.601 (1.075–2.384)	0.404	0.785	1.743 (1.232–2.465)	0.701	0.806	1.426 (0.930–2.188)	0.949	0.746

## Data Availability

The data presented in this study are available on request from the corresponding author. The data are not publicly available due to hospital policy.
